# Analysis of Superjunction MOSFET (CoolMOS^TM^) Concept Limitations—Part II: Simulations

**DOI:** 10.3390/ma18235468

**Published:** 2025-12-04

**Authors:** Zbigniew Lisik, Jacek Podgórski

**Affiliations:** Department of Semiconductor and Optoelectronic Devices, Lodz University of Technology, Aleje Politechniki 8, 93-590 Lodz, Poland

**Keywords:** modeling, MOS devices, power MOSFETs, power semiconductor devices, power electronics

## Abstract

The CoolMOS^TM^ (Infineon Technologies AG, Munich, Germany) has been regarded as a device that alleviates high-voltage limitations of unipolar power devices. However, although the theoretical considerations seem to confirm this possibility, this expectation has not been fulfilled to date. It appears that there are some limitations in the CoolMOS^TM^ concept, and the paper deals with their identification. Part I concentrated on the theory of high-voltage superjunction and its implementation into a power VDMOS transistor, which resulted in the CoolMOS^TM^ structure. This part is aimed at the physical and technological limitations that have been identified, taking advantage of numerical investigations of CoolMOS^TM^ structures developed on the basis of a typical VDMOS one.

## 1. Introduction

**Figure 1:**
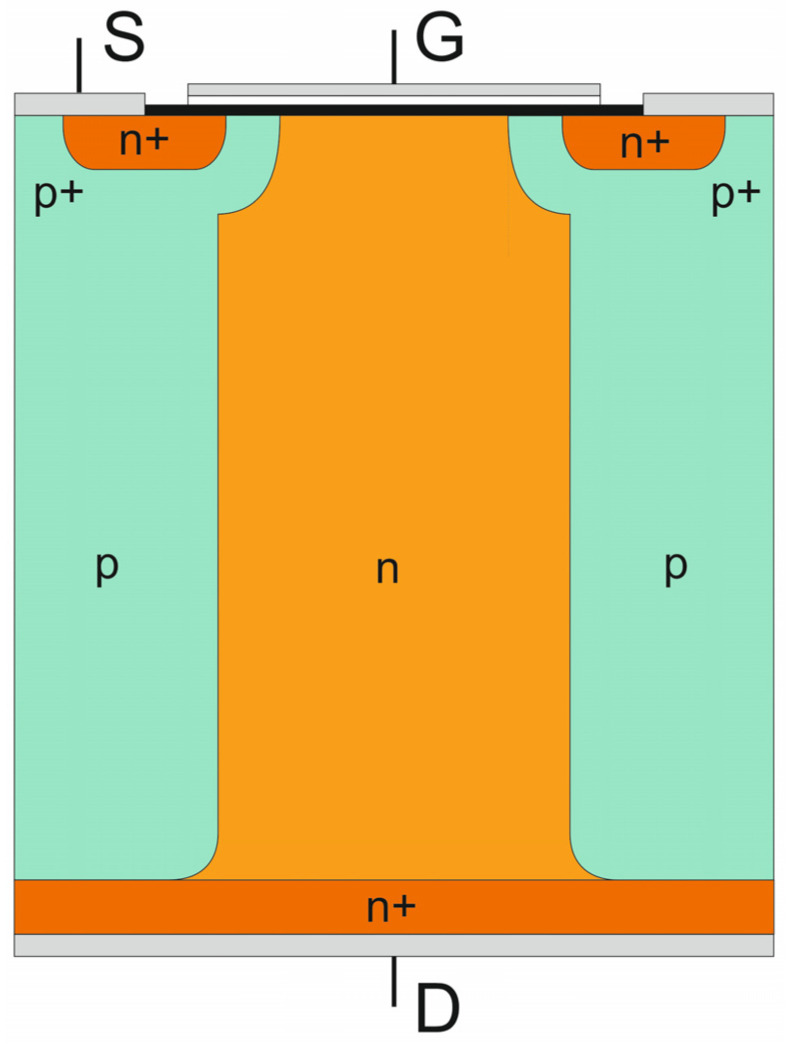
Basic cell of CoolMOS^TM^ transistor.

The concept of the CoolMOS^TM^ transistor is based on the conventional MOSFET principle, supplemented by the superjunction idea applied to the reconstruction of its high-voltage junction. It is described in Part I [1] of the article, which is focused on theoretical considerations to gain insight into the physical phenomena within superjunction structures and to identify any physical constraints in the superjunction concept. As a result, the first coherent presentation of the concept evolution from its physical background to its practical applications has been created. The single transistor cell under consideration is shown in Figure 1. It consists of one central n-doped column surrounded by two p-doped ones characterized by the same doping level, while the high-voltage junction plane is partially vertical rather than purely horizontal.

The prospective advantage of the CoolMOS^TM^ construction over the VDMOS one is presented in many publications, e.g., in [2,3], and seems to offer almost unlimited possibilities for the increase in blocking voltage. Though this idea was very promising, the rating parameters of the first devices introduced to the market in 1999, have not been as attractive as one could expect. Since that time, their construction has been the subject of many investigations dealing with super-junction application. There were theoretical approaches using analytical or numerical tools [4,5,6,7] as well as experimental works [8,9,10,11]. Till now, however, their maximum blocking voltage has not exceeded 800 V.

One can suppose that it results from the fact that, although the CoolMOS^TM^ basics are known, the phenomena taking place inside manufactured devices limit their real possibilities. Unfortunately, this problem has so far only been mentioned in the literature and has not been the subject of a serious published investigation. The paper aims to gain a deeper understanding of these phenomena, especially with respect to their occurrence in the vicinity of fabrication divergences, in comparison to the CoolMOS^TM^ concept. Part I focuses on the theoretical considerations aimed at identifying any physical constraints in the superjunction concept, while Part II presents the results of numerical investigations addressing the influence of divergences on the performance of real devices. Taking advantage of Sentaurus TCAD (Synopsys Inc. Mountain View, CA, USA) [12], they used the 2D computer model presented below for the simulations. Their introductory results have already been partially shown in [13,14]. The full numerical analysis of the problem, presenting both the constraints resulting from the superjunction idea as well as any consequences resulting from discrepancies in the CoolMOS^TM^ transistor design and fabrication, is presented in the paper.

## 2. Materials and Methods

### 2.1. Numerical Models

The advantages of the CoolMOS^TM^ concept are limited by theoretical constraints presented in Part I; however, they need not be kept in the real manufactured devices due to physical or technological obstacles. The influence of such discrepancies can be easily evaluated using numerical investigations, allowing more precise analysis of the phenomena inside a real CoolMOS^TM^ structure.

**Figure 2:**
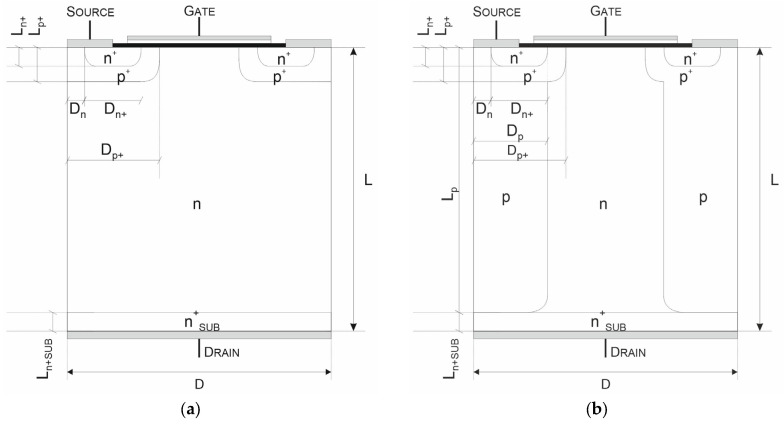
Cross-section of the reference VDMOS (**a**) and basic COOLMOS (**b**) structures.

Such investigations have been performed in [14], taking a typical VDMOS structure, shown in Figure 2a, as the reference. To obtain a representative device for consideration, its parameters were compiled as an average based on a comprehensive literature review [15,16,17,18,19,20,21,22,23,24] and are presented in Table 1. They were used to establish the design of the counterpart COOLMOS-1 structure that is depicted in Figure 2b. Its parameters, fixed by adoption of the VDMOS ones, are presented in Table 1. It was used as the basic one in the carried out numerical investigations.

During device simulations, Sentaurus TCAD ver. U-2022.12 was used to determine the electrical characteristics, as well as distributions of carriers, potentials, electric fields, and currents. The SRH model was used to describe trap-assisted recombination, the Auger model was employed to account for non-radiative recombination processes, and the van Overstraeten model specifically modeled avalanche (impact ionization) breakdown. Additionally, carrier mobility models dependent on doping concentration and electric field were included, together with effects related to effective intrinsic carrier density and bandgap narrowing.

All simulations were conducted at a temperature of 300 K for all structures, assuming ideal ohmic contacts. Limitations of these simulations include the 2D nature of the model, neglect of thermal effects, and simplified treatment of surface interface states, which could influence real device performance. Despite these limitations, the simulations provide valuable insights into current flow distribution, parasitic JFET effects, and space charge region formation, which are difficult to measure experimentally.

The results obtained from these simulations were used to evaluate the impact of structural parameters such as n- and p-pillar widths, doping concentrations, and pillar alignment on key electrical characteristics, including on-state resistance (R_ON_) and breakdown voltage (V_B_). This approach allowed a systematic analysis of the trade-offs involved in CoolMOS™ design and provided guidance for optimizing both the superjunction and MOS control parts of the device.

### 2.2. Simulation Results—Reference VDMOS vs. COOLMOS Counterparts

The results of numerical simulations aimed at the evaluation of the breakdown voltage V_B_ and the on-state resistance R_ON_ of VDMOS and its counterpart COOLMOS-1 are presented in Table 2. They show that the introduction of a p-pillar alone into the reference VDMOS results in a significant increase in the breakdown voltage, but it is accompanied by an increase in on-resistance, which becomes unacceptably large. However, it can be easily reduced by decreasing the device thickness L. Reducing it to 40 µm produced a new structure called COOLMOS-2, with the same breakdown voltage as the reference VDMOS and a lower, although still a bit larger, on-resistance.

According to (17) [1] in Part I, the on-resistance can also be reduced by increasing the dopant concentration in the pillars. However, it must be performed according to the constraints presented in Part I, particularly those shown in Figure 9 [1], which defines the superjunction mode area (SJM) in the (w,N) domain. If the device is to remain an SJ one, at the pillar width 30 µm, the doping concentration in the pillars must be lower than 1.25 · 10^15^ cm^−3^. This was carried out in the subsequent test structure, COOLMOS-3, with a pillar doping concentration of only 1 · 10^15^ cm^−3^. Its parameters, also presented in Table 2, are better than those of the reference VDMOS. Its on-resistance decreased to 0.177 Ωcm^2^ at a blocking voltage of 1004 V. The blocking voltage is lower than in the COOLMOS-1 structure due to the increase in the threshold electric field E_thn_ according to (13) in Part I. It is worth noticing that when other parameters are fixed, its magnitude is limited to the order of 1000 V.

The above results show that, due to the theoretical constraints indicated in Part I, a direct transformation of a VDMOS structure into a CoolMOS^TM^ device, keeping the design parameters unchanged, does not produce a mature device; only after a simple modification of a single parameter in the SJ high-voltage part can it result in a power transistor characterized by the expected low on-resistance and very high blocking voltage. It seems that some progress will be possible when the modifications are more comprehensive, covering both the superjunction and MOS control parts. In particular, they should take into account the physical or technological limitations presented below.

## 3. Discussion

### 3.1. JFET Effect

**Figure 3:**
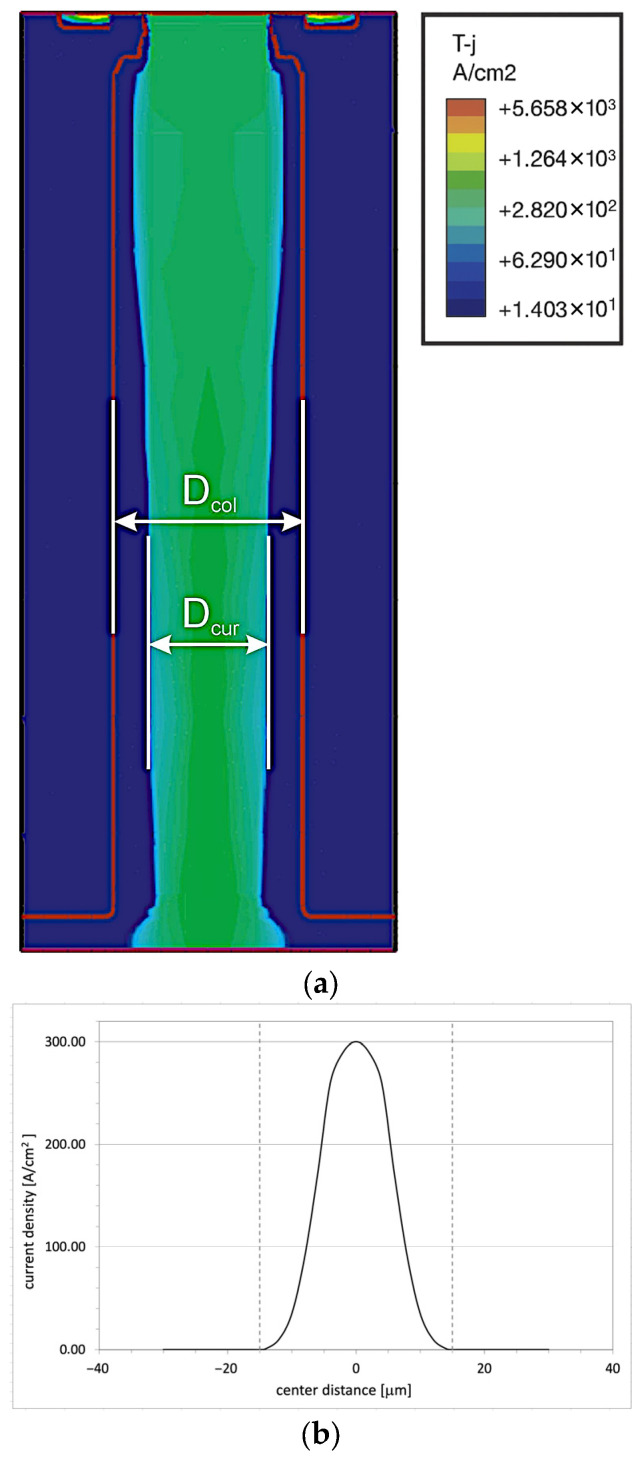
Parasitic JFET effect in conducting COOLMOS transistor: (**a**) current distribution in the n-column, (**b**) current density distribution in the n-column cross-section.

In VDMOS transistors, the forward current flows homogenously through low-doped n-layers, whereas in COOLMOS devices, the introduction of SJ limits the area of its flow to the n-pillars only. If the only difference between the two transistors is the introduction of a meander-shaped junction created by a set of symmetric p and n pillars, as it takes place in the COOLMOS-1 structure, one can expect the COOLMOS-1 on-resistance to be exactly twice as large, due to the current path being twice as narrow. The simulation has not confirmed that, and the difference between the on-resistances presented in Table 2 is much larger. The reason for the above discrepancy was the subject of numerical investigations taking advantage of the COOLMOS-1 numerical model. They allowed evaluating the 2D distribution of current flow presented in Figure 3. Contrary to the theoretical consideration in Part I, the path of current flow D_cur_ is narrower than the n-pillar D_col,_ whereas the remaining area of the n-pillar is occupied by the p-n space charge region DSCR, according to relation (1). Additionally, the current flow is not homogeneous, but the cross-section of its density is described by a bell-shaped function shown in Figure 3. Both phenomena are responsible for much larger on-resistance in the COOLMOS-1 structure.

It is the consequence of the fact that the n-pillar is limited by p–n junctions and acts as a channel of some parasitic JFET consisting of the n-pillar channel layer and two p-gate layers created by adjacent p-pillars. It is controlled by the reverse bias of gate–channel junctions, whose magnitude is equal to the voltage drop on the induced channel in the MOS transistor component. The dimension of the current path in n-pillar D_cur_ depends on several factors. First, it is limited by the SCR region that is created in any JFET structure due to:

- Built-in diffusion potential at the p–n plane, which requires an SCR layer with a thickness of D_SCR_(0) ≈ 6 nm on the n-pillar side of the investigated COOLMOS-1 structure;
- External reverse bias from the voltage drop across the MOS transistor, which causes expansion of the SCR to D_SCR_(MOS) ≈ 2.8 µm in the investigated COOLMOS-1 structure;
- Squeezing of the current path, as shown in Figure 3, leading to an increase in the SCR layer along the channel, since the voltage drop accompanying the current flow in the channel increases the local reverse polarization. In the investigated COOLMOS-1 structure, the additional increase in SCR layer at the drain was D_SCR_(JFET) ≈ 8.5 µm.

All the above phenomena can cause an increase in COOLMOS on-resistance in comparison with its theoretical evaluation presented in Part I. The increase can be reduced by the increase in donor concentration in the n-pillar, but it must keep the restriction presented in Figure 9 in Part I. As long as the final D_SCR_ is kept significantly smaller than D_col_, like in the case of COOLMOS-1, the device will act properly, although with noticeably worse on-resistance.

(1) $$D_{cur}=D_{col}-D_{SCR}$$

According to (1), the n-pillar dimension D_col_ is the second factor influencing the current path D_cur_. At a constant magnitude of the SCR layer D_SCR_, its decrease diminishes the part of the pillar occupied by the current, thereby lowering the total on-resistance of the COOLMOS structure. It can be described by the coefficient of effective channel width:

(2) $$d=\frac{D_{cur}}{D_{col}}\cdot 100\%$$

**Figure 4:**
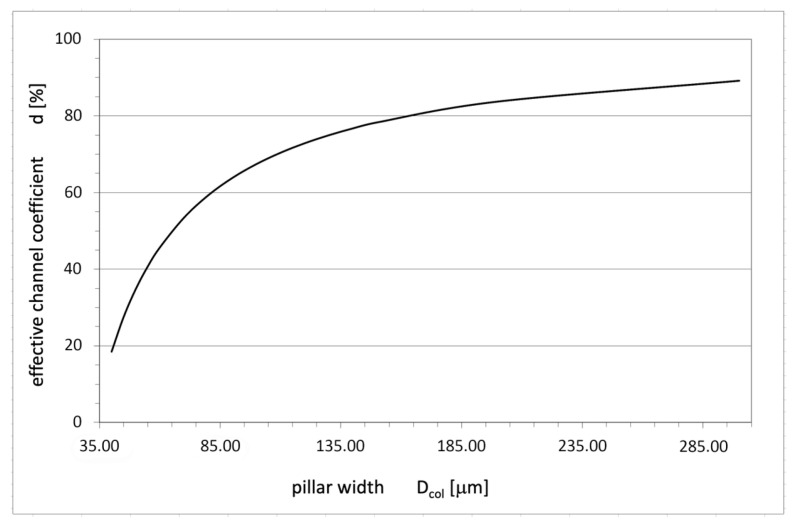
Coefficient of effective channel width d dependence on the pillar width D_col_.

The simulation performed for COOLMOS-1 structures with different magnitudes of width D and corresponding changes in pillar dimensions allowed determining the dependence of coefficient d on the structure width D. As shown in Figure 4, the current path is drastically reduced, resulting in a corresponding increase in on-resistance for thin n-pillars. It is an additional physical limitation for SJ design that must be considered together with the limitations stemming from Figure 9 in Part I.

### 3.2. Technological Limitations

**Figure 5:**
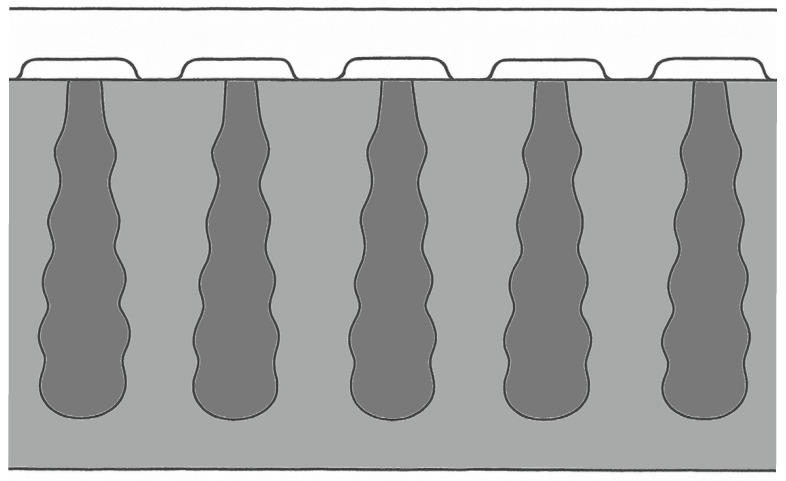
A schematic illustration showing the non-uniformity of the columnar structure intended for the superjunction, formed during successive epitaxial processes.

The symmetry of doping concentration and thickness of the n- and p-pillars creating the high-voltage SJ, as well as the parallelism of the pillar edges, are considered elements of SJ theory presented in Part I. It is obvious, however, that due to technological reasons, it is almost impossible to keep both parameters under such conditions. The doping density can differ a little between two doping processes, and the fabrication of a straight-line junction edge, typically using a several-step epitaxy process, is practically impossible. A schematic illustration reflecting the shape of the columns for the superjunction, as actually formed during the multi-step epitaxial process, is shown in Figure 5.

**Figure 6:**
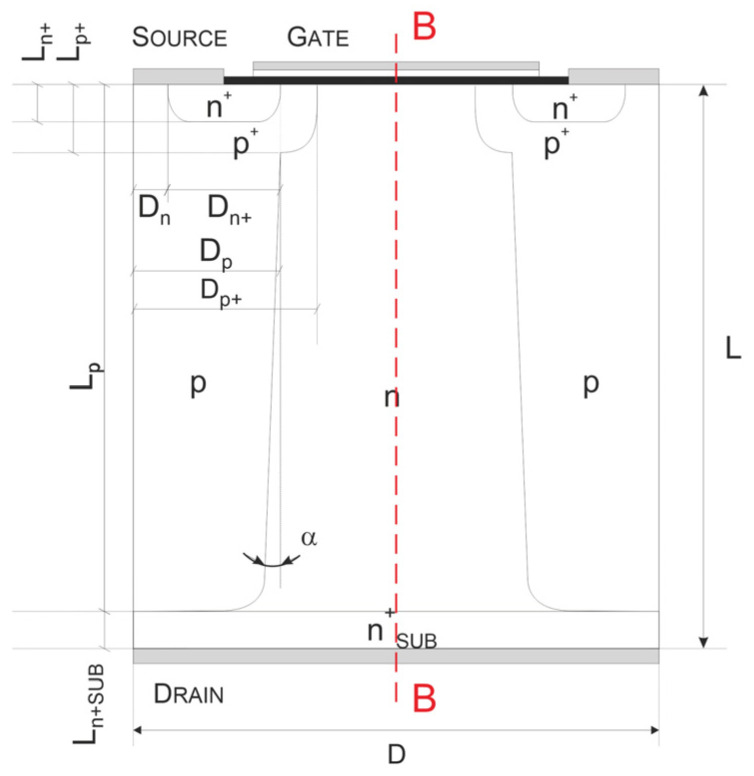
Cross-section of the considered COOLMOS structure.

As one can notice, the edges are significantly inclined, which can influence the COOLMOS feature. To study the influence of the mentioned incorrectness of these construction parameters on the relationship between on-resistance R_ON_ and blocking voltage V_B_, 2D simulations using the COOLMOS-1 numerical model were performed for a slightly modified structure, as shown in Figure 6. It covers the additional possibility to incline the pillar edges by the angle α that in the normal simulation is equal to 0.

**Figure 7:**
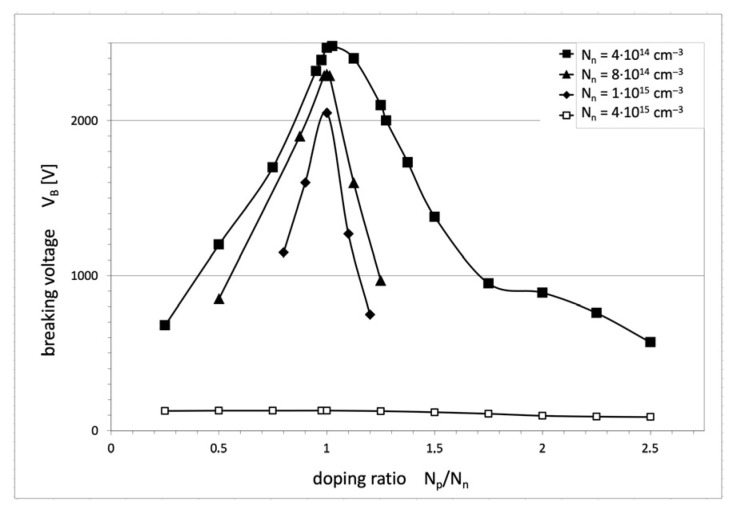
Dependence of the breakdown voltage V_B_ vs. the doping ratio N_p_/N_n_ in superjunction columns for different doping levels in the n-column.

In Part I, it has been stated that the optimal design of COOLMOS must follow the relation (14) [1], which means that when the widths of n- and p-pillars are the same, the doping concentration in the pillars should also be the same, and any discrepancies in this matter would result in worse voltage V_B_ breakdown. The results of numerical investigations performed for COOLMOS-1 structures with different levels of n-pillar dopant concentration confirm that. They are shown in Figure 7, presenting the dependence of breakdown voltage V_B_ on the doping ratio N_p_/N_n_ for different magnitudes of n-pillar doping. The presented curves prove that even a small deviation from the doping equilibrium rule leads to a drastic decrease in the maximum blocking voltage V_B_, especially for the high doping level, which is necessary to obtain a low on-resistance. In the figure, the maximal breakdown voltage decreases with the increase of N_n_ doping, which is in good agreement with the theoretical considerations in Part I.

**Figure 8:**
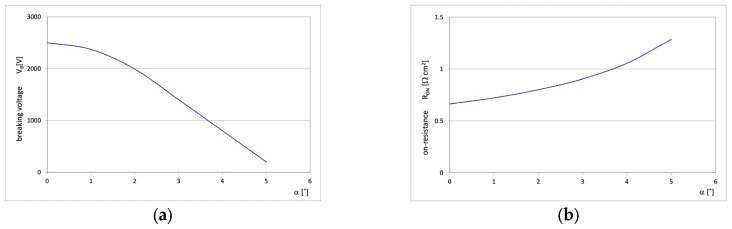
Dependence of the breakdown voltage V_B_ (**a**) and on-resistance R_ON_ (**b**) vs. the angle α.

The influence of n-pillar deviation from the vertical direction has been examined using the numerical model developed for the structure shown in Figure 6. The simulation indicated that even a little deviation from the vertical direction leads to a rapid decrease in breakdown voltage and a significant increase in on-resistance. It is shown in more detail in Figure 8, presenting the changes in both the parameters when the angle α between the pillar edge and the vertical axis changes from 0 to only 5°. Even such small changes in the edge slip result in drastic changes in the breakdown voltage and the on-resistance.

**Figure 9:**
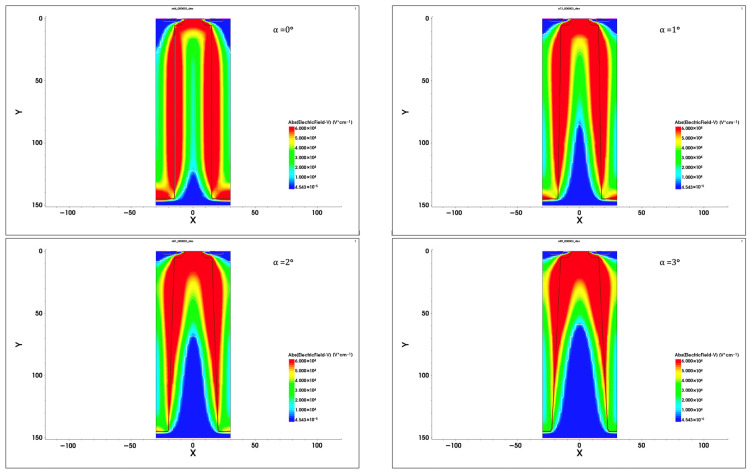
Two-dimensional maps of the electric field in the n-pillar of the investigated structure for increasing angle α (white contour represents the SCR border).

**Figure 10:**
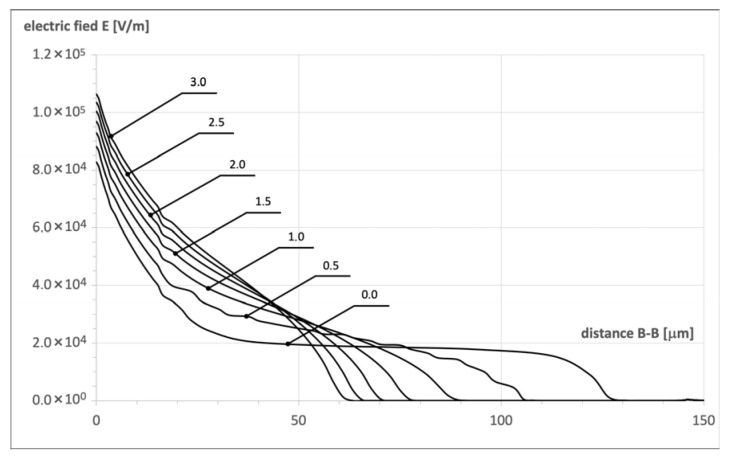
Electric field strength distributions in n-pillar along axis B–B in Figure 6 for different angle α magnitudes (V_DS_ = 300 V).

It is the consequence of disturbances in the superjunction effect (shown in Figure 7 in Part I), which are illustrated in Figure 9. It covers the series of 2D maps presenting the electric field distribution in the n-pillar of the investigated structure for increasing angle α. The white contour represents the borders of SCR filling the pillar, which is pushed out when the angle α increases, and in this way, the SJ effects gradually disappear. It is noticed more clearly in Figure 10, presenting the changes in electric field strength along the axis B–B in Figure 6. When α = 0°, the flat electric field distribution occurs in the whole pillar, and, according to (15) in Part I, the breakdown voltage increases without any limits following the increase in pillar length. When α rises, the SCR shape changes and transforms into a triangle that occupies the lower part of the n-pillar. The distance m in (15) in Part I is shorter, and the second term in the equation becomes negligibly small. This way, the SJ effect disappears, and the magnitude of breakdown voltage becomes equal to its magnitude in the case of a planar p–n junction.

The findings provide practical guidance: achieving low R_ON_ while maintaining high V_B_ requires a compromise between doping levels, pillar width, and structural symmetry. Designers must account for parasitic JFET effects and possible misalignments to optimize performance in real CoolMOS^TM^ devices.

The analysis highlights practical design implications for CoolMOS^TM^ devices. Achieving low on-resistance (R_ON_) while maintaining high breakdown voltage (V_B_) requires careful optimization of pillar dimensions, doping levels, and structural symmetry. Narrow n-pillars increase the impact of parasitic JFET effects, reducing the effective current path and increasing R_ON_, while misalignments or deviations from ideal pillar geometry strongly reduce V_B_. The SCR expansion, influenced by both voltage drop along the channel and reverse bias of junctions, further limits current conduction. A simple correlation analysis of the simulated structures shows that a 10% decrease in pillar width can increase R_ON_ by up to 20%, while even a 2° deviation of the pillar angle can reduce V_B_ by nearly 15%. These results emphasize the sensitivity of CoolMOS^TM^ performance to small geometrical and doping variations. Designers must therefore consider these interactions holistically, balancing doping concentration, pillar width, and junction alignment to achieve the desired performance.

## 4. Conclusions

The CoolMOS^TM^ has been considered a device that alleviates high-voltage limitations of unipolar power devices. However, the theoretical considerations seem to confirm such a possibility; this expectation has not been fulfilled till now. The initial expectations turned out to be too optimistic, and there are some limitations in the CoolMOS^TM^ concept. The paper deals with their identification.

Part I [1] has focused on theoretical limitations both in the case of the superjunction concept alone and in the case of the CoolMOS^TM^ structure. It has been proven that the superjunction effect is not an inherent feature of SJ diode design. At low reverse voltage it works as an ordinary p–n diode and starts to work in the superjunction mode (SJM) only when some restrictions are fulfilled, e.g., the electric field strength reaches the magnitude of threshold electrical field Eth and the pillar symmetry defined by (14) in Part I occurs.

The numerical investigations in this Part have been focused on the physical and technological limitations of the superjunction in CoolMOS^TM^ transistors, which narrows down the theoretical constraints mentioned in Part I. First, the presence of the parasitic JFET structure was considered. It is formed by the n-pillar, which acts as the channel with a changeable effective width, and two adjacent p-pillars as the p-gates. The simulation proves that the effective channel is drastically diminished by the increase in SCR layer in the n-pillar due to the built-in diffusion potential at the p-n plane, external reverse bias due to the voltage drop on the MOS transistor, and the squeezing of the current path due to the voltage drop along the path of the current flow. It leads to an undesirable increase in on-resistance and can even lead to blocking of the current flow if the width of the n-pillar is too small.

The symmetry of doping concentration and thickness of the n- and p-pillars as well as the parallelism of the pillar edges, constitute inherent elements of high-voltage SJ theory presented in Part I. Its parameters can be, however, sensitive to the discrepancy from the ideal SJ concept. It has been tested by numerical simulations. The results presented in Figure 7 show that even a small deviation from the doping equilibrium rules leads to a drastic decrease in the maximum blocking voltage V_B_. A similar effect has been noticed in the case of inclined pillar edges. The simulations indicated that even a little deviation from the vertical direction, even below 5°, leads to a rapid decrease in breakdown voltage and a significant increase in on-resistance, as it is shown in Figure 8. It is the consequence of the disruption of the flat-field domain along the pillar due to the changes in its width, as it is shown in Figure 9.

The results presented in the paper allow us to conclude that the previously suggested prospective advantages of CoolMOS^TM^ have been too optimistic, and presently, one cannot realistically expect new CoolMOS^TM^ devices to combine high blocking voltage with reasonably low on-resistance. The same theoretical, physical, and technological limitations that make the design and fabrication of the device difficult must be taken into account. One can expect, however, that with sufficient effort to address the identified constraints, it will be possible to develop such devices.

## Figures and Tables

**Table 1:** Basic parameters of the considered COOLMOS-1 and VDMOS reference structure.

	Parameter	VDMOS	COOLMOS-1
doping [cm^−3^]	n^+^	1 · 10^20^	1 · 10^20^
	p^+^	1 · 10^16^	1 · 10^16^
	p	-	4 · 10^14^
	n	4 · 10^14^	4 · 10^14^
	n^+^_SUB_	5 · 10^16^	45 · 10^16^
dimensions [μm]	L	150	150
	D	60	60
	L_n+_	2	2
	L_p+_	5	5
	L_p_	-	145
	L_n_^+^_SUB_	5	5
	D_n_	7	7
	D_n+_	8	8
	D_p+_	19	19
	D_p_	-	15
	α	-	0

**Table 2:** Considered parameters of reference VDMOS structure and its successive counterpart, COOLMOS structures.

	VDMOS	COOLMOS-1	COOLMOS-2	COOLMOS-3
V_B_ [V]	500	2500	500	1004
R_ON_ [Ωcm^2^]	0.201	0.660	0.211	0.177
L [µm]	150	150	40	150
N_n_ = N_p_ [cm^−3^]	4.0 · 10^14^	4.0 · 10^14^	4.0 · 10^14^	1.0 · 10^15^

## Data Availability

The original contributions presented in this study are included in the article. Further inquiries can be directed to the corresponding author.
